# The concentration of testosterone, pituitary adenylate cyclase-activating polypeptide, and protamine 1 in the serum of male chicken following administration of epididymis and testicular extracts and their combination

**DOI:** 10.14202/vetworld.2019.1101-1107

**Published:** 2019-07-25

**Authors:** Muslim Akmal, Gholib Gholib, Rinidar Rinidar, Fitriani Fitriani, T. Zahrial Helmi, Sugito Sugito, M. Isa, Nurliana Nurliana, Sri Wahyuni, Dasrul Dasrul, M. Aman Yaman

**Affiliations:** 1 Laboratory of Histology, Faculty of Veterinary Medicine, Universitas Syiah Kuala, Banda Aceh, Aceh, Indonesia; 2 Laboratory of Physiology, Faculty of Veterinary Medicine, Universitas Syiah Kuala, Banda Aceh, Aceh, Indonesia; 3 Laboratory of Pharmacology, Faculty of Veterinary Medicine, Universitas Syiah Kuala, Banda Aceh, Aceh, Indonesia; 4 Laboratory of Biochemistry, Faculty of Veterinary Medicine, Universitas Syiah Kuala, Banda Aceh, Aceh, Indonesia; 5 Laboratory of Clinic, Faculty of Veterinary Medicine, Universitas Syiah Kuala, Banda Aceh, Aceh, Indonesia; 6 Laboratory of Veterinary Public Health, Faculty of Veterinary Medicine, Universitas Syiah Kuala, Banda Aceh, Aceh, Indonesia; 7 Laboratory of Anatomy, Faculty of Veterinary Medicine, Universitas Syiah Kuala, Banda Aceh, Aceh, Indonesia; 8 Laboratory of Reproduction, Faculty of Veterinary Medicine, Universitas Syiah Kuala, Banda Aceh, Aceh, Indonesia; 9 Field Laboratory of Animal Sciences, Faculty of Veterinary Medicine, Universitas Syiah Kuala, Banda Aceh, Aceh, Indonesia

**Keywords:** chicken, epididymis and testicular extracts, pituitary adenylate cyclase-activating polypeptide, spermatogenesis, testosterone, protamine 1

## Abstract

**Bakcground and Aim:**

Testis and epididymis are male reproductive organs that play an important role in spermatogenesis**.** These two organs are rich in the content of hormones and other molecules needed in the process of spermatogenesis which affect the quality of the spermatozoa. The objective of this study was to examine the effect of the administration of epididymis and testicular extracts and their combination on testosterone, pituitary adenylate cyclase-activating polypeptide (PACAP), and protamine 1 (PRM1) concentrations in the serum of male chicken.

**Materials and Methods:**

Twenty male chickens (broiler strain Cp707), aged 3 weeks and weighing 800-1000 g, were randomly divided into four different groups including a control group (T0) = injected with 1 ml normal saline and treatment groups: T1 = injected with 1 ml epididymis extract, T2 = injected with 1 ml testicular extract, and T3 = injected with a combination of 1 ml epididymis + 1 ml testicular extract. The experiment was conducted for 13 days and at the end of the study (day 14), the chickens were sacrificed to obtain the serum. Furthermore, the concentrations of testosterone, PACAP, and PRM1 were then measured by using an enzyme-linked immunosorbent assay technique.

**Results:**

The concentrations of PACAP and PRM1 did not show a significant difference between treatment groups (T1, T2, and T3) and control group (T0) (p>0.05). However, the concentration of testosterone showed a significantly higher difference in a group injected with a combination of 1 ml epididymis and 1 ml testicular extracts (T3) compared to the control group (T0) (p<0.05).

**Conclusion:**

The administration of epididymis and testicular extracts and their combination did not affect the increase of PACAP and PRM1 concentration. However, a combination of these extracts significantly affects the increase of testosterone concentration in the serum of male chicken.

## Introduction

Spermatogenesis is a complex biological process involving genetics [1] and hormonal factors, especially testosterone [2]. Testosterone is an essential steroid hormone for spermatogenesis. In Sertoli cells, when testosterone bond with androgen receptor (AR), it will induce two signaling pathways namely MAP kinase and Ca^2+^ pathways. Both of the pathways will induce the phosphorylation of cyclic adenosine monophosphate (cAMP) response element-binding protein (CREB) [3]. Moreover, testosterone also induces the cAMP-responsive element modulator (CREM) in the testicular tissue [4]. In chicken–pheasant hybrids, low testosterone levels cause deterioration of steroid biosynthetic activity in the Leydig cells, which affects the absence of secondary sexual characteristics and disruption of spermatogenesis [5]. In addition to testosterone, pituitary adenylate cyclase-activating polypeptide (PACAP) is an essential molecule in spermatogenesis and steroidogenesis [6]. This molecule is expressed significantly in the brain, testis, and spermatids [7,8]. Previous studies on various species reported that PACAP has an important role in the hormonal control of spermatogenesis [8] and steroidogenesis [8,9] and the growth and activation of spermatozoa [10]. Another study reported that PACAP is involved in stimulating the secretion of testosterone in the culture of Leydig cells [11].

Protamine (PRM) also has an important role in spermatogenesis and the maintenance of sperm quality [12]. Several studies showed that deficiency of PRM resulted in sperm DNA damage [13], fertilization and embryo development disorders [14], and male fertility [15]. Moreover, it leads to a decline in spermatozoa quality [16]. Another study showed that deficiency of PRM1 expression generated damage of sperm chromatin structure [17].

Our previous study showed that the administration of epididymis extract increased the concentration of testosterone [18], estrogen [19], and the quality of sperm in local male goats [20]. We believe that this result was caused by an action of molecules, particularly PACAP and/or testosterone contained in the epididymis extract. Reglodi *et al*. [21] revealed that PACAP is present at high concentration in the testis. Lv *et al*. [22] showed that epididymis and testis are the organs rich in PACAP expression.

So far, the effect of epididymis and testicular extracts on spermatogenesis in chicken is absent. Therefore, in the present study, we experimented to examine the effect of administration of epididymis and testicular extracts and their combination on the testosterone, PACAP, and PRM1 concentrations in the serum of male chicken.

## Materials and Methods

### Ethical approval

The Committee of Animal Ethics of Faculty of Veterinary Medicine, Universitas Syiah Kuala, Banda Aceh, approved this study (Ref: 17/KEPH/IX/2018).

### Extraction of epididymis and testes

The preparation of epididymis and testicular extracts was performed as described by Akmal *et al*. [23]. In brief, epididymis and testes of local adult goats (bucks) were collected from the slaughterhouse. The age of the bucks ranged from 1.5 to 2 years, with a body weight of 14-16 kg. They do not exhibit a breeding season, but they can breed throughout the year. The epididymis and testes were then transported to the Laboratory of Histology, Faculty of Veterinary Medicine, Universitas Syiah Kuala, Banda Aceh. The samples were then soaked into water for simplifying in the dissociation of the epididymis and testes. Furthermore, the samples were sliced to a smaller size and weighed. After that, they were ground and added with 10 ml aquadestilata per gram of epididymis and testes; the resultant obtained was sieved with a filter paper. The mixture solution was then centrifugated at 3000 rpm for 20 min. Finally, the supernatant was taken and stored at −20°C in the freezer before use.

### Treatments

This study used twenty male chickens (broiler, strain Cp707), aged 3 weeks and weighed 800-1000 g. Prior to the study, the chickens were acclimatized for 7 days in a new environment to avert stress. The chickens were then randomly divided into four groups (five chickens per group) including a control group (T0) and three treatment groups (T1, T2, and T3). The control group (T0) was injected with 1 ml normal saline, and the treatment groups were injected with 1 ml epididymis extract (T1), 1 ml testicular extract (T2), and a combination of 1 ml epididymis extract + 1 ml testicular extract (T3). The extracts were induced through intramuscular administration. The experiment was conducted for 13 days according to our previous study [18]. During the study, the chickens were fed *ad libitum*. At the end of the study, blood was collected from the brachial wing vein and then processed as serum for testosterone, PACAP, and PRM1 measurements. After that, the chickens were sacrificed for meat consumption.

### Measurement of testosterone

The concentration of testosterone was measured using a testosterone enzyme-linked immunosorbent assay (ELISA) (Cat no. EIA-1559, DRG Instruments GmbH, Germany). Measurement of testosterone was performed as described by Gholib *et al*. [24]. In brief, duplicate 25-μl aliquots of serum were assayed along with 25 μl aliquot standard (dose range 0.2-16 ng/ml) and control on microtiter plates coated with a monoclonal mouse antibody of testosterone. Afterward, 200-μl enzyme conjugate was added to each well (except the blank well), was sealed with adhesive tapes, and was incubated for 60 min at room temperature. Following incubation, the plates were then washed four times with washing solution and blotted dry. Furthermore, 200-μl substrate solution (tetramethylbenzidine) was added to each well and was sealed with adhesive tapes. The plates were then re-incubated for 20 min at room temperature. The enzyme reaction was stopped with 100-μl stop solution in each well. Finally, absorbance was measured at 450 nm using a plate reader (ELISA reader). The testosterone was calculated using the Microplate Manager-6 (MPM-6) Software (Bio-Rad Laboratories, Inc., USA).

### Measurement of PACAP

The concentration of PACAP was measured using a chicken PACAP ELISA (Cat no. MBS269153, My BioSource.com). Measurement of PACAP was performed following the manufacturer’s instruction. In brief, duplicate 100-μl aliquots of serum were assayed along with 100-μl aliquots of chicken PACAP standard (dose range 0.312-20 ng/ml) and control on microtiter plates coated with a chicken PACAP monoclonal antibody. The microtiter plates were then sealed with adhesive tapes and incubated in an incubator at 37°C for 90 min. Following incubation, the plates were then washed two times with washing solution and blotted dry. After that, 100-μl biotinylated chicken PACAP antibody was added to each well. The microtiter plates were sealed with adhesive tapes, and re-incubated in an incubator at 37°C for 60 min. The plates were then rewashed three times using a washing solution and blotted dry. Furthermore, 100-μl enzyme conjugate was added to each well except blank wells. The microtiter plates were sealed with adhesive tapes, and re-incubated in an incubator at 37°C for 30 min. The plates were then washed 5 times using a washing solution. After that, 100-μl substrate solution (tetramethylbenzidine) was added to each well (also into the blank well) and was re-incubated in a dark incubator at 37°C for 30 min. After that, the enzyme reaction was stopped with 100-μl stop solution and mixed well. Finally, absorbance was measured at 450 nm using an automatic plate reader (ELISA reader). The concentration of chicken PACAP was then calculated using the Microplate Manager-6 (MPM-6) Software (Bio-Rad Laboratories, Inc., USA).

### Measurement of chicken PRM1

The concentration of PRM1 was measured using a chicken PRM1 ELISA (Cat no. MBS264971, My BioSource.com). Measurement of PRM1 was performed following the manufacturer’s instruction. In brief, duplicate 100-μl aliquots of serum were assayed along with 100-μl aliquots of chicken PRM1 standard (dose range 0.156-10 ng/ml) and control on microtiter plates coated with a chicken PRM1 monoclonal antibody. The microtiter plates weresealed with adhesive tapes and incubated in an incubator at 37°C for 90 min. Following incubation, the plates were washed two times with washing solution and blotted dry. After that, 100-μl biotinylated chicken PRM1 antibody was then added to each well. The microtiter plates were sealed with adhesive tapes, and re-incubated in an incubator at 37°C for 60 min. The plates were then rewashed three times using a washing solution and blotted dry. Furthermore, 100-μl enzyme conjugate was added to each well except blank wells, The microtiter plates were sealed with adhesive tapes, and re-incubated in an incubator at 37°C for 30 min. The plates were then washed 5 times using washing solution. After that, 100-μl substrate solution (tetramethylbenzidine) was added to each well (also into the blank well) and re-incubated in a dark incubator at 37°C for 30 min. After that, the enzyme reaction was stopped with 100-μl stop solution and mixed well. Finally, absorbance was measured at 450 nm using an automatic plate reader (ELISA reader). The concentration of chicken PRM1 was then calculated using the Microplate Manager-6 (MPM-6) Software (Bio-Rad Laboratories, Inc., USA).

### Statistical analysis

Data were analyzed using one-way analysis of variance (ANOVA) followed by *post hoc* analysis using Duncan’s multiple range test. A statistical significance was set to α=0.05, and statistical analysis was conducted using SPSS 20 (IBM, USA).

## Results

**Figure-1:**
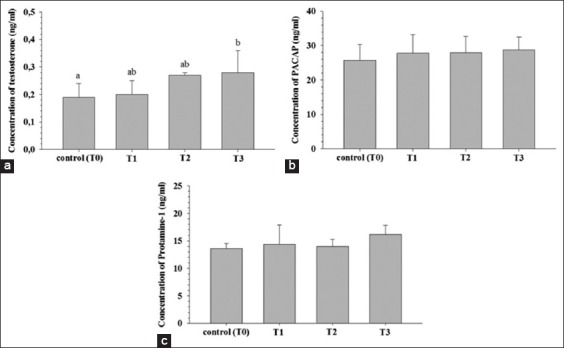
The concentration (mean ± standard deviation) of testosterone (a), pituitary adenylate cyclase-activating polypeptide (b), and protamine 1 (c) after injected with epididymis and testicular extracts and their combination in male chicken. Different superscripts indicate a statistically significant difference between groups (p<0.05).

Administration of epididymis and testicular extracts and their combination increased the concentration of testosterone, PACAP, and PRM1 in male chicken. The mean (±standard deviation) of testosterone, PACAP, and PRM1 concentration in the serum of male chickens is presented in Table-1 and Figure-1.

**Table 1:** The concentration of testosterone, PACAP, and PRM1 (mean±standard deviation) after injected with epididymis and testicular extracts and their combination in male chicken.

Treatments	Testosterone ng/ml	PACAP ng/ml	PRM1 ng/ml
T0	0.19±0.05	25.72±4.60	13.60±0.89
T1	0.20±0.05	27.78±5.47	14.40±3.50
T2	0.27±0.01	27.90±4.80	14.00±1.22
T3	0.28±0.08	28.80±3.63	16.20±1.64

Annotation: T0=Chickens were injected with 1ml normal saline, T1=Chickens were injected with 1mlepididymis extract, T2=Chickens were injected with 1ml testicular extract, T3=Chickens were injected with a combination of 1 ml epididymis+1 ml testicular extracts. PACAP=Pituitary adenylate cyclase-activating polypeptide, PRM1=Protamine 1

The concentration of testosterone in the treatment groups (T1, T2, and T3) showed an increase 5.26%, 42.11%, and 47.37%, respectively, compared to the control group (T0) (Table-1). Based on ANOVA results, the concentration of testosterone showed a significant difference between groups (Figure-1a). *Post hoc* analysis using Duncan’s multiple range test showed that the concentration of testosterone in group T3 was significantly higher compared to the control group (T0) (p<0.05). However, testosterone concentration of groups T1 and T2 was not significantly different compared to that of the control groups (T0) (p>0.05).

The concentration of PACAP in the serum of the treatment groups (T1, T2, and T3) showed an increase (8.01-9.98%) when compared to that of the control group (T0) (Table-1 and Figure-1b). However, based on ANOVA results, there was no significant difference in the PACAP concentration between the control group and treatment groups (p>0.05).

In addition to testosterone and PACAP, the concentration of PRM1 in the treatment groups (T1, T2, and T3) was higher (5.88%, 2.94%, and 19.12%, respectively) compared to that of the control group (T0) (Table-1 and Figure-1c). However, based on ANOVA results, there was no significant difference in the PRM1 concentration between the control group and treatment groups (p>0.05).

## Discussion

The present study demonstrates the importance of utilization of epididymis and testicular extracts and their combination to stimulate the concentration of testosterone, PACAP, and PRM1 in male chicken. The results showed that testosterone concentrations were significantly increased by 47.37% after administration of the combination of epididymis and testicular extracts. These findings indicate that epididymis and testicular extracts are a useful source for improving male gonadal hormone, especially testosterone. Testosterone is a crucial steroid hormone for male reproductive development and function [25]. It is very important for maintaining spermatogenesis process [26] and male fertility [27].

For spermatogenesis, the bond between testosterone and its receptor (AR) can induce two testosterone signaling pathways, namely MAP kinase and calcium (Ca^2+^). These two pathways phosphorylate CREB and CREB-mediated gene expression in the nucleus of Sertoli cells [3]. A previous study also showed that in Sertoli cells, testosterone can induce the expression of CREM in testicular cells [4]. From this study, it seems that the administration of a combination of the epididymis and testicular extracts can stimulate epididymis and testes to increase the activation of cAMP production [28] and induce steroidogenesis in Leydig cells [29]. Similar results were reported by Yuliansyah *et al*. [18] and Akmal *et al*. [19] that administration of epididymis extract increased testosterone and estrogen concentrations and the quality of sperm in local male goats [20].

CREB and CREM are important molecules for spermatogenesis [30,31]. They play an important role as transducers of hormonal signals into the induction of gene expression [31] and a master regulator of molecular process for all stages of spermatogenesis [32]. In humans, disturbances in CREB expression result in azoospermia [30]. Another study showed that CREM is essential for the differentiation of post-meiotic germ cells to stimulate the action of hormones in regulating spermatogenesis genes [33]. In addition, CREM is very important for spermatid development and maturation in rodents, monkeys, primates, and humans [34], and it is an essential factor for spermiogenesis [35]. CREM arranges the expression of some important post-meiosis genes such as transitional protein genes and PRMs [33].

In contrast to testosterone, the concentrations of PACAP and PRM1 were not significantly increased after administration of the epididymis and testicular extracts, or their combination.. Nevertheless, the PACAP and PRM1 concentrations in the treatment groups tended to increase by 8.01-9.98% and 2.94-19.12% respectively, compared to those of the control group. A study showed that epididymis and testes are a source of PACAP [36,37]. For example, in the testis, the expression of PACAP mRNA is first seen on day 20 after birth, and it reaches a maximum level on day 60. It is especially expressed in the spermatocytes and round spermatids, whereas in the epididymis, the expression of PACAP mRNA is first seen on day 10 after birth, and it reaches maximum levels from day 40 [22].

PACAP is a neuropeptide that has a pivotal role in inducing the diversity of biological effects [37]. In bird testis, PACAP is a great candidate which acts as a regulator of Leydig cell function [9] to secrete testosterone hormone [38]. Many evidences have shown that PACAP has an essential role in reproductive endocrinology [10], in the control of mammalian spermatogenesis [6], and in the motility of human sperm cells [10].

PRMs are nuclear proteins expressed in haploid male germ cells [39], which are essential for male fertility [40]. For vertebrate sperm, there are two distinct types of PRMs namely PRM1 and PRM2 which are covering the DNA in mammalian sperm [41]. In roosters, PRM mRNA was expressed significantly in round spermatid and elongated spermatid [42]. PRM1 was expressed in the sperm of every species [43], whereas DNA in rodent and primate sperm was packaged by PRM1 and PRM2 [44]. PRM acts as an important contributor in spermatozoa quality, successful fertilization, development of preimplantation embryos [45], and male fertility [46]. Our previous study on mice showed that the disturbances of PRM2 expression influence the fertilization ability and the number of child production [12]. The results showed that PRMs in birds might be related to internal fertilization and egg laying [47].

In concordance with our results, the administration of these extracts did not affect the increase of PRM1, but testosterone did. This result suggests that PRM1 concentrations are not completely arranged by the testosterone through the CREM signaling pathway. It indicates that mechanism of molecular signaling pathway in spermatogenesis is complex which needs to be investigated in future studies. Nevertheless, from this study, we believe that the combination of these extracts may be useful for inducing spermatogenesis in male chicken.

## Conclusion

The administration of epididymis and testicular extracts and their combination in male chicken did not affect the increase of PACAP and PRM1 concentrations. However, the combination of epididymis and testicular extracts affects significantly on the increase of testosterone concentrations in the serum of male chicken.

## Authors’ Contributions

MA, SW, and NN designed the experiments. MA, MI, and DD performed the experiments. FF and RR extracted the epididymis and testes. GG measured the concentration of PACAP, testosterone, and PRM1. MA, GG, and SW analyzed the data. MA, GG, and SS wrote the manuscript. TZH and MAY provided logistical support for the experiments. All authors read and approved the final manuscript.
